# Are Current or Future Mesothelioma Epidemics in Hong Kong the Tragic Legacy of Uncontrolled Use of Asbestos in the Past?

**DOI:** 10.1289/ehp.0900868

**Published:** 2009-10-22

**Authors:** Lap Ah Tse, Ignatius Tak-sun Yu, William Goggins, Mark Clements, Xiao Rong Wang, Joseph Siu-kie Au, Kai Shing Yu

**Affiliations:** 1 School of Public Health and Primary Care, Chinese University of Hong Kong, Hong Kong, China; 2 National Centre for Epidemiology and Population Health, Australian National University, Canberra, Australia; 3 Department of Clinical Oncology, Queen Elizabeth Hospital, Kowloon, Hong Kong SAR, China

**Keywords:** asbestos, epidemic, incidence, mesothelioma, time trends

## Abstract

**Background:**

Because of the long latent period of asbestos-related mesothelioma, investigators suggest that the high incidence of this disease will continue in the coming decades.

**Objectives:**

We describe the time trends of mesothelioma incidence and its relationship to historical consumption of asbestos in Hong Kong and project future trends of mesothelioma incidence.

**Methods:**

We obtained local annual consumption of total asbestos for 1960–2006 (converted to kilograms per person per year). Age-standardized incidence rates (ASIRs) of mesothelioma were computed and depicted on graphs using the centered moving average method. Indirectly standardized rates were regressed on a transformation of consumption data that assumed that the latency between asbestos exposure and mesothelioma diagnosis followed a normal distribution with a mean ± SD of 42 ± 10.5 years.

**Results:**

ASIRs for males started to increase substantially in 1994 and were highest in 2004; for females, ASIRs climbed in the 1980s and in the early 1990s but have fluctuated without obvious trends in recent years. The highest asbestos consumption level in Hong Kong was in 1960–1963 and then decreased sharply afterward. Using past asbestos consumption patterns, we predict that the mesothelioma incidence rate for males will peak in 2009, with the number of cases peaking in 2014, and then slowly decline in the coming decades.

**Conclusions:**

Hong Kong experienced an epidemic of mesothelioma from 2000 to 2006 that corresponded with the peak of local asbestos consumption in the early 1960s assuming an average latent period of 42 years. The incidence is anticipated to decline in the coming decades but may not decrease back to the background risk level (the risk unrelated to asbestos exposure).

Globally, malignant mesothelioma was a rare neoplasm before the 1950s, but its incidence has been increasing steeply since the 1970s in many parts of the world (Hemminki and Li 2003; Leigh and Driscoll 2003; Peto et al. 1999; Price and Ware 2004; Ulvestad et al. 2003). Occupational exposure to asbestos is considered to be the most important risk factor for malignant mesothelioma; it is responsible for 70–83% of the risk for males and 38% for females (Chang et al. 2006; Goldberg et al. 2006; Hemminki and Li 2003). Other causes implicated in the etiology of mesothelioma include radiotherapy, erionite fibers, and chronic inflammation (Huncharek 2002; Neumann et al. 2001). The average latent period between initial exposure to asbestos and the onset of mesothelioma is approximately 40 years and may range up to 60 years depending on the level of lifelong exposure (Chang et al. 2006; Hyland et al. 2007; Peto et al. 1999). Despite the existence of country-to-country disparities in mesothelioma trends due to variations in asbestos use, the universally long latent period dictates that the high incidence and mortality rates of mesothelioma will continue in the coming decades (Nishikawa et al. 2008; Peto et al. 1999).

Hong Kong is not an area known for producing asbestos or asbestos products, but historically, it had used asbestos extensively. Since the 1950s, local annual consumption of asbestos started to increase in response to the booming economic development, especially in the dockyard and construction industries, with its use peaking in the early 1960s (Lam et al. 1983). As in most other places in the world (Nishikawa et al. 2008; Peto et al. 1999), Hong Kong might experience an epidemic of mesothelioma as a consequence of the uncontrolled use of asbestos in the past decades. In this article, we describe the time trends of mesothelioma incidence and its relationship to the historical consumption of asbestos and project the future trend of mesothelioma incidence in Hong Kong.

## Materials and Methods

### Source of incidence data

We obtained the data used in this study from the Hong Kong Cancer Registry, which continuously collects cancer information by checking the computerized databases and the medical records of all public and most private hospitals and pathology laboratories. The incidence data from this source are considered to be representative for the territory with the maximum coverage (Tse et al. 2009), and the extracted data contained new primary malignant mesothelioma cases diagnosed during the period 1983–2006. Only three male cases of malignant mesothelioma were observed in the Hong Kong population before 1983 (1976, 1978 and 1982), and no cases were reported among females (Lam et al. 1983). All malignant mesothelioma cases during the study period were coded as C45 according to the *International Classification of Diseases, 10th Revision* [World Health Organization (WHO) 2004].

Population estimates during the corresponding period and population projections were obtained from the Hong Kong Census and Statistics Department (Census and Statistics Department 2009a). We used mid-year population data to calculate the incidence rate.

### Local asbestos consumption

We obtained information on the annual import, export, and reexport of all types of asbestos from the Hong Kong Census and Statistics Department. Local annual consumption of total asbestos (all types) was estimated using three indices: total import, total import minus export, and total import minus export and reexport (Census and Statistics Department 2009b); these asbestos exposure indices were converted to per capita asbestos use (expressed as kilograms per person per year) by dividing the total consumption by the size of the population for the corresponding year. Per capita asbestos use was documented as a more useful surrogate for measuring the general asbestos exposure level of a population (Lin et al. 2007; Nishikawa et al. 2008).

### Statistical analyses

We standardized incidence rates by the direct method using the WHO 1966 world standard population as the reference (Ahmad et al. 2001). Using the centered moving average method with a 5-year interval, we depicted in graphs the time trends of age-standardized incidence rate (ASIR) of mesothelioma, as well as the historical trends of annual per capita asbestos use in terms of three asbestos indices: total import, total import minus reexport, and total import minus export and reexport.

To project the future trends of the occurrence of mesothelioma for males, we assumed that the latency time from asbestos exposure to mesothelioma diagnosis followed a normal distribution with a mean of 42 years, which was based on a study by Lin et al. (2007), and a standard deviation of 10.5 years from an Australian study (Clements et al. 2007). We recognized that these figures might not exactly describe the latency distribution in Hong Kong. However, we believed they were reasonable. Adopting these figures was further supported by a study by Chang et al. (2006), which found a mean latency of 46 years and a standard deviation (SD) of 11 years for a very small number (*n* = 22) of Hong Kong mesothelioma patients with available dates of first asbestos exposure. We transformed the asbestos consumption data using the convolution


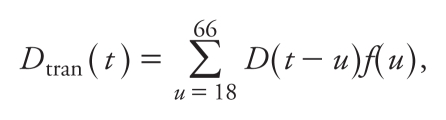


where *D*_tran_(*t*) represents the local annual consumption of total asbestos in year *t*, and *f*(*u*) follows a normal (42 ± 10.5 years) distribution. When transforming the asbestos exposure data, we assumed that asbestos exposure before 1960 was negligible and that asbestos exposure after 2006 remained at 2006 levels. We also assumed that the contribution from latency periods of < 18 years or > 66 years was negligible. The model was fitted using each index of local annual consumption of total asbestos (i.e., total import, total import minus reexport, or total import minus export and reexport); the model fits were very similar, and we used total imports as the exposure measure for future predictions. Because too few cases were available, we did not develop a model for female mesothelioma. In addition, the number of male cases was small, so our model used indirectly standardized rates, where the expected numbers of cases were calculated using the standard age-specific rates for the period 1972–2006 and population estimates or population projections. We used Poisson regression with the observed numbers of cases as the outcome, with an offset that was the sum of the log of the expected number of cases (indirect standardization) and the log of *D*_tran_(*t*). The model had the following form: mean of cases = (expected cases) × *D*_tran_(*t*) × exp(β), where β was to be estimated.

## Results

### Trends in incidence

During the period 1976–2006, a total of 199 new cases of mesothelioma were diagnosed (137 males and 62 females) in the general population of Hong Kong (Hong Kong Cancer Registry 2010).

Table 1 summarizes the incidence of mesothelioma by age and calendar year for males and females. The number of mesothelioma cases rose sharply from 1972–1976 to 2002–2006, especially for males. The highest rates were generally observed for both males and females ≥ 70 years of age, whereas the younger age groups consistently had lower rates during the study period.

**Figure 1:**
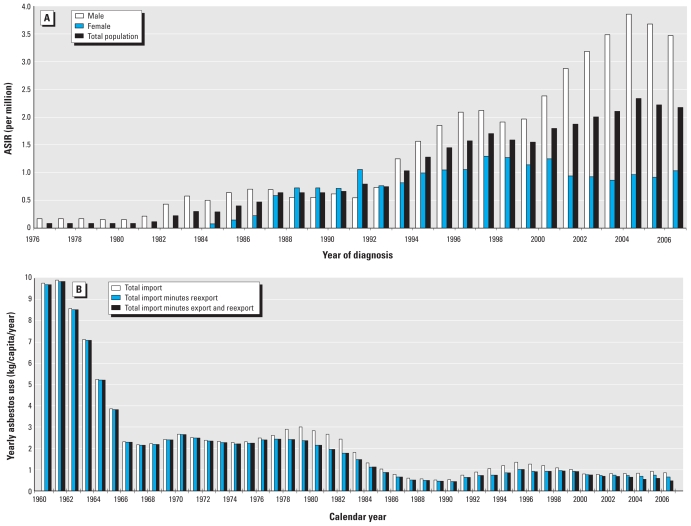
(*A*) ASIRs of mesothelioma among adults in the Hong Kong general population during 1976–2006, using centered moving average method by a 5-year interval (no cases were reported for females before 1986). (*B*) Annual per capita asbestos use (kg/capita/year) in Hong Kong during 1960–2006 (total import, total import minus reexport, and total import minus export and reexport), using centered moving average method, by a 5-year interval.

Figure 1A shows the time trends of ASIRs of mesothelioma in the Hong Kong general population, based on the centered moving average method with a 5-year interval. Before 1993–1994, the moving average of ASIRs for males was relatively low (0.16–0.73/million); it started to increase in 1993–1994 (1.25–1.56/million), peaked in 2004 (3.86/million), and then decreased slightly to 3.47/million in 2006. A similar increasing trend was also observed for females that began earlier but became less remarkable in more recent years, which has resulted in an increasing male-to-female ratio from 0.82 in 1987–1991 to 3.77 in 2002–2006. For males and females, the combined moving-average ASIR trend first peaked in 1997 at 1.71/million, decreased slightly in the next 2 years, and then climbed to the highest peak in 2004 at around 2.34/million.

### Trends in asbestos consumption

Trade statistics of asbestos use were available only from 1960 (Figure 1B). During 1960–2006, the average annual asbestos use per capita for total import, total import minus reexport, and total import minus export and reexport in Hong Kong was 2.34 kg, 2.17 kg, and 2.15 kg, respectively. The highest asbestos consumption level in Hong Kong was in 1960–1963, with an average use of 8.84 kg/capita/year (equivalent to a total consumption of > 30 × 10^6^ kg annually). The average use was lower, at 2.61 kg/capita/year, for total import during 1964–1984 (equivalent to an annual consumption of 9.7 × 10^6^ kg) and fell further afterward, but a slight rebound was noted in the mid-1990s. From 1960 to 2006, local annual per capita use of asbestos reduced by 93.5% for total import, 96% for total import minus reexport, and 98.5% for total import minus export and reexport, respectively.

### Projections for 2007–2025

**Figure 2:**
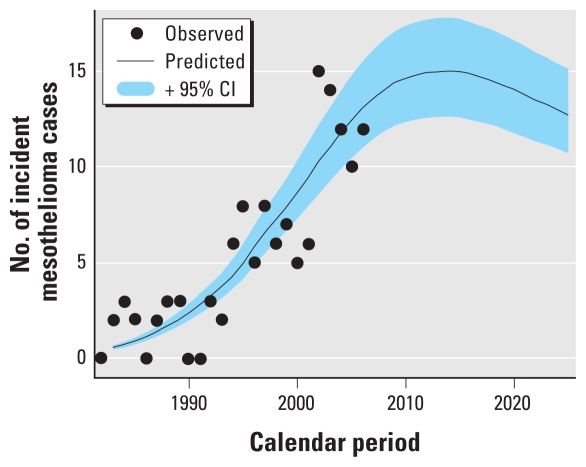
Projections for the predicted numbers of mesothelioma cases for males in the Hong Kong general population for 2002–2027, assuming a normally distributed latency period of 42 ± 10.5 years (mean ± SD).

We undertook model fitting and projections for males only because the number of females (*n* = 62) with mesothelioma was too small. Assuming a latency distribution with a mean of 42 years and a SD of ± 10.5 years for mesothelioma, we estimated the intercept term as −0.277 [95% confidence interval (CI), −0.447 to −0.1079]. The projections indicated that the indirectly standardized rates for incident mesothelioma cases for males would peak in 2009, whereas the number of cases would peak at 15 cases/year in 2014, assuming asbestos consumption remains at 2006 levels for the near future (Figure 2).

## Discussion

We observed a notably increasing trend in ASIRs for malignant mesothelioma among Hong Kong males from 1976–2006; the increasing trend was initially apparent for females, but there was no evidence for its continuation in recent years. Males and females ≥ 70 years of age had the highest incidence rates during the study period. Based on past asbestos consumption patterns, we predict that the mesothelioma incidence rate among men will peak in 2009, with the number of cases peaking in 2014, and then will slowly decline in the coming decades.

The increasing trend of mesothelioma incidence that Hong Kong has experienced in the past few decades is similar to that observed in Western European countries (France, Germany, Britian, Italy, Netherlands, and Switzerland) (Peto et al. 1999), Norway (Ulvestad et al. 2003), Australia (Leigh and Driscoll 2003), and Japan (Kanazawa et al. 2006). The United States has reported a leveling off in incidence rates for males for approximately 10 years. This finding suggests that the peak has already passed (Price and Ware 2004). On the other hand, the peak for many South American countries (e.g., Brazil) is expected to occur in the 2010s or 2020s, due to the later ban of asbestos use (Nishikawa et al. 2008; Pedra et al. 2008).

Mesothelioma shows a strong male predominance and can be explained by the male-dominated asbestos-related occupations such as asbestos mining and shipyard work [International Agency for Research on Cancer (IARC) 1987]. In Hong Kong, asbestos consumption began to rise in the 1950s (Lam et al. 1983), with the highest peak in the early 1960s. Given an average latency period of 42 years (± 10.5 years), the local peak use of asbestos in the early 1960s would result in a projected peak mesothelioma incidence rate in 2009 and peak number of cases in 2014, with the number of cases and incidence rate falling to relatively low levels after the rapid decline of asbestos use in the 1990s and 2000s especially after the enactment of legislation prohibiting the sale and import of blue asbestos (crocidolite) and brown asbestos (amosite) in Hong Kong in 1996 (Environmental Hong Kong 2003). One investigator estimated that locally consumed products containing commercial asbestos types were broadly in the ratio of 70–80% chrysotile to 20–30% amphiboles (crocidolite or amosite) before 1996 (Tsin TW, personal communication). However, chrysotile asbestos continued to be used commercially in various industries in Hong Kong after 1996. The annual consumption of asbestos (mainly chrysotile) rebounded to a higher level in the mid-1990s, and the consequence on the future occurrence of mesothelioma will need to be monitored.

Excess risk of malignant mesothelioma is associated with shipyard work and other forms of asbestos exposures, including household contacts with asbestos workers (IARC 1987). Amphiboles, such as crocidolite and amosite, were generally believed to be more potent than was chrysotile in inducing mesothelioma (Berman and Crump 2008; Hodgson and Darnton 2000). However, existing evidence from both animal and human studies support a link between chrysotile asbestos and malignant mesothelioma (Langer and McCaughey 1982; Rogers et al. 1991). A rat inhalation study observed an excess risk of mesothelioma for those exposed to Zimbabwean chrysotile (Lippmann 1994). Case-report and case–control studies showed that the lungs of mesothelioma patients contained only chrysotile fibers rather than amphiboles (Langer and McCaughey 1982; Rogers et al. 1991). Two recent studies provided further supportive evidence for a positive association between chrysotile and mesothelioma (Lippmann 1994; Yano et al. 2009). If the link between chrysotile and mesothelioma does exist, the mesothelioma epidemic would be expected to continue in Hong Kong in the future, as a consequence of the ongoing use of chrysotile asbestos.

Interestingly, the average asbestos consumption for 1960 and 1970 in Hong Kong was 6.14 kg/capita/year, which was higher than any country reported by Lin et al. (2007). The booming economy and explosive population growth during the early 1960s might explain the high consumption of asbestos at that time. In the late 1950s and the early 1960s, Hong Kong began building massive public housing projects to accommodate the influx of hundreds of thousands of refugees from mainland China after the Great Leap Forward (a political movement launched by Mao Zedong, the former chairman of the Chinese Communist Party) during 1958–1960 and the associated nationwide “Three Years of Natural Disasters” between 1958 and 1961 (Chen 2009). An urban renewal strategic study conducted by the Hong Kong Urban Renewal Authority (Planning and Lands Branch 2009) revealed that most buildings ≥ 30 old were in unsatisfactory condition and did not satisfy modern fire safety building design and installation requirements. Individual prewar buildings without modern sanitary facilities were prioritized for renewal actions, including redevelopment, eradicating run-down buildings, and restructuring. A documented 8,500 buildings were ≥ 30 years old in the Hong Kong metro area in 1998, but the number will double to 16,000 by 2016 (Planning and Lands Branch 2009). Asbestos fibers previously locked in the buildings (e.g., roof, fireproofing brick, and sound isolation materials) will be released into the environment through the renewal works, which creates possibilities for continuous secondary exposures in the environment. Even if effective control measures and/or tighter legislative controls are put in place immediately regarding import and use of asbestos products, previous use of asbestos will result in the accumulation of asbestos in the environment, potentially increasing the risk of mesothelioma in the community. An estimated 38% of malignant mesothelioma can be attributed to such environmental exposures (Agudo et al. 2000). A more recent study in Japan demonstrated that neighborhood exposure to asbestos could pose a serious risk to both male (standardized mortality ratio = 13.9; 95% CI, 5.6–28.7) and female residents (standardized mortality ratio = 41.1; 95% CI, 15.2–90.1) across a wide area (Kurumatani and Kumagai 2008).

Changes in diagnostic criteria may influence trends of mesothelioma incidence but should not be a major issue in our study, because the proportion of morphologically verified cases in Hong Kong has been stable (≥ 85%) in recent decades (Hong Kong Cancer Registry 2008). However, lack of data on local asbestos consumption before 1960 made it difficult to analyze the full consequences of asbestos exposure. Moreover, we recognize that no distinction was available among asbestos fiber types in the available trade statistics, which posed another limitation for this study.

In conclusion, Hong Kong has been experiencing an epidemic of mesothelioma since 2000, which well parallels the peak of local asbestos consumption in the early 1960s. Although the mesothelioma incidence is anticipated to decline in the coming decades, it may not decrease to background risk levels given that chrysotile consumption has not been banned under the current legislation and that secondary asbestos exposure from the environment will likely continue. Nevertheless, the hypotheses generated from this ecologic study need further confirmation by subsequent analytic studies. The present study provides supportive evidence for an immediate and global ban on asbestos use.

## Figures and Tables

**Table 1:** Mesothelioma incident cases and ASIRs (per million), by sex and calendar year (5-year interval) in Hong Kong, 1972–2006.

	Age group (years)						
Calendar year	< 40	40–49	50–59	60–69	70–79	≥ 80	Total^a^
Males							
1972–1976	0.12 (1)^b^	0 (0)	0 (0)	0 (0)	0 (0)	0 (0)	0.09 (1)
1977–1981	0 (0)	0 (0)	0 (0)	1.38 (1)	0 (0)	0 (0)	0.08 (1)
1982–1986	0.30 (3)	0.70 (1)	1.47 (2)	0 (0)	5.50 (2)	0 (0)	0.57 (8)
1987–1991	0.20 (2)	1.23 (2)	0.71 (1)	0.97 (1)	4.17 (2)	0 (0)	0.55 (8)
1992–1993	0.10 (1)	2.24 (5)	3.64 (5)	3.36 (4)	11.69 (7)	12.35 (2)	1.57 (24)
1997–2001	0.11 (1)	0.35 (1)	4.29 (7)	6.05 (8)	16.96 (13)	8.73 (2)	1.96 (32)
2002–2006	0.35 (3)	1.27 (4)	6.08 (13)	10.28 (13)	26.02 (24)	18.47 (6)	3.86 (63)
Total	0.05 (11)	0.29 (13)	1.29 (28)	1.87 (27)	6.77 (48)	6.17 (10)	0.63 (137)
Females							
1972–1976	0 (0)	0 (0)	0 (0)	0 (0)	0 (0)	0 (0)	0 (0)
1977–1981	0 (0)	0 (0)	0 (0)	0 (0)	0 (0)	0 (0)	0 (0)
1982–1986	0 (0)	0 (0)	0 (0)	1.09 (1)	0 (0)	0 (0)	0.08 (1)
1987–1991	0.32 (3)	0 (0)	1.63 (2)	0.00 (0)	4.91 (3)	8.01 (2)	0.72 (10)
1992–1993	0.62 (6)	0.99 (2)	0.87 (1)	2.58 (3)	2.71 (2)	3.14 (1)	1.00 (15)
1997–2001	0.20 (2)	1.07 (3)	2.12 (3)	4.08 (5)	5.60 (5)	2.38 (1)	1.14 (19)
2002–2006	0.21 (2)	0.88 (3)	1.45 (3)	3.46 (4)	2.95 (3)	3.47 (2)	0.97 (17)
Total	0.21 (13)	0.61 (8)	0.98 (9)	1.88 (13)	2.85 (13)	2.95 (6)	0.64 (62)

a Crude incidence rate.

b Numbers in parentheses are new cases of mesothelioma.
