# Erratum

**Published:** 2010-06

The Editor’s Summary for the article “Are Current or Future Mesothelioma Epidemics in Hong Kong the Tragic Legacy of Uncontrolled Use of Asbestos in the Past?,” by Tse et al. [ Environ Health Perspect 118:382–386 (2010); doi:10.1289/ehp.090086], has been corrected online: specifically, “(which has been implicated but not conclusively established as a cause of mesothelioma)” has been deleted. The corrected summary appears below.

Inhaled asbestos fibers may contribute to three-fourths of malignant mesotheliomas diagnosed in men and almost 40% of cases diagnosed in women. Bans on the manufacture and sale of amphibole asbestos fibers are expected to reduce the incidence of mesothelioma, but the long latency period from initial exposure to clinical disease means that people exposed before bans were enacted will continue to develop asbestos-related mesotheliomas as they age. Tse et al. (p. 382) used historical data on asbestos consumption and mesothelioma diagnoses to predict future mesothelioma trends in Hong Kong. Asbestos use peaked during a construction boom in the early 1960s and subsequently declined by > 90% following a ban on the sale and import of crocidolite and amosite asbestos in 1996, whereas mesothelioma diagnoses in men increased from a single case in 1972–1976 to 63 cases in 2002–2006 (corresponding to crude incidence rates of 0.09 and 3.86 cases/million men, respectively). Assuming an average latency of 42 years, the authors predict that incidence rates will peak in 2009 and that diagnoses will peak in 2014. However, they caution that ongoing use of chrysotile asbestos and the release of asbestos fibers from older buildings during demolition or renovation may slow the projected decline.

